# The Impact of Lignin Structural Diversity on Performance of Cellulose Nanofiber (CNF)-Starch Composite Films

**DOI:** 10.3390/polym11030538

**Published:** 2019-03-21

**Authors:** Yadong Zhao, Ayumu Tagami, Galina Dobele, Mikael E. Lindström, Olena Sevastyanova

**Affiliations:** 1 Department of Fibre and Polymer Technology, KTH Royal Institute of Technology, Teknikringen 56-58, 100 44 Stockholm, Sweden; ayumu@kth.se (A.T.); mil@kth.se (M.E.L.); 2 Research Laboratory, Nippon Paper Industries Co., Ltd., 5-21-1 Oji, Kita-ku, Tokyo 114-0002, Japan; 3 Latvian State Institute of Wood Chemistry, 27 Dzerbenes Str., LV 1006 Riga, Latvia; gdobele@edi.lv; 4 WWSC, KTH Royal Institute of Technology, Teknikringen 56-58, 100 44 Stockholm, Sweden

**Keywords:** lignin, successive solvent fractionation, tunicate cellulose nanofibers-starch-lignin composites, film properties, interrelation

## Abstract

Lignin fractions having different molecular weights and varied chemical structures isolated from kraft lignins of both softwood and hardwood via a sequential solvent fractionation technique were incorporated into a tunicate cellulose nanofibers (CNF)—starch mixture to prepare 100% bio-based composite films. The aim was to investigate the impact of lignin structural diversity on film performance. It was confirmed that lignin’s distribution in the films was dependent on the polarity of solvents used for fractionation (acetone > methanol > ethanol > ethyl acetate) and influenced the optical properties of the films. The –OH group content and molecular weight of lignin were positively related to film density. In general, the addition of lignin fractions led to decrease in thermal stability and increase in Young’s modulus of the composite films. The modulus of the films was found to decrease as the molecular weight of lignin increased, and a higher amount of carboxyl and phenolic –OH groups in the lignin fraction resulted in films with higher stiffness. The thermal analysis showed higher char content formation for lignin-containing films in a nitrogen atmosphere with increased molecular weight. In an oxygen atmosphere, the phenol content, saturated side chains and short chain structures of lignin had impacts on the maximum decomposition temperature of the films, confirming the relationship between the chemical structure of lignin and thermo-oxidative stability of the corresponding film. This study addresses the importance of lignin diversities on composite film performance, which could be helpful for tailoring lignin’s applications in bio-based materials based on their specific characteristics.

## 1. Introduction

Plastic is a highly useful and convenient material. Although the plastic production benefits the daily life of society, 90% of the plastic products were used once and then discarded, thus creating a global environmental crisis [1]. Recently, sustainable materials produced from renewable resources have obtained a lot of attention to replace the petroleum-based plastic materials [2]. By following up this trend, the bio-based composites are considered to be the next generation materials to replace the conventional petroleum-based composites in numerous application fields, such as green packaging for food, biodegradable hydrogel for wound dressing, bio-sensors for clinical diagnosis, cellulose-based materials for water purification [3,4,5] and barrier properties [6] after certain chemical modifications [7,8,9] and aerogel for environmental pollution absorption [10]. The production of bio-based materials in 2011 was 3.5 billion tons, and it is expected to boost to 12 billion tons in 2020 [11]. In order to meet the expanding market, both industry and academia are putting efforts to create new bio-based materials made of bio-polymers from forest, agricultural and food industries: cellulose, collagen, starch, chitosan, alginate, gelatin, etc. [12].

Lignin is one of such polymers and is the second most abundant biopolymer after cellulose and the most abundant aromatic biopolymers in nature. It is generated in large quantities as the main by-product from the pulp and paper industries. Commercially, 120 Mt of kraft pulp is produced annually, and this corresponds to a total production of 70 Mt of kraft lignin [13,14]. Recently, the development of both second-generation biofuel techniques and new biorefineries for numerous biomass types has further boosted lignin production capacity. To broaden lignin applications, intensive efforts have been taken to develop its uses as an adhesive, sorbent, carbon fiber, resin, biologically active agent carrier and animal feed additive [15]. However, the majority of lignin from the pulp and paper industry meets with low value utilization, namely, burning for energy, and only 2% is used commercially [16]. More recently, the interest in using lignin as an added-value compound for thermoplastic or packaging film by developing sustainable composites using different kinds of matrices has arisen. By taking advantages of the polymeric nature, functionality, thermal stability, hydrophobicity, antioxidant and antimicrobial properties, lignin has been incorporated into different matrixes to fabricate functional biocomposites, such as in films with hydroxypropylcellulose [17], cellulose [18], polypropylene [19], starch [20] and polyolefins [21]. In cellulose nanofibril composite, the Colloidal Lignin Particles (CLPs) improves the ductility and mechanical strength due to their lubricating and stress transferring effects [22]. The antioxidant ability of lignin provides to the chitosan film a radical scavenging activity, essentially governed by a surface activity mechanism [3]. As one natural UV-protective polymer, the addition of lignin into poly(lactic-acid) and gelatin composite films has proven to provide the UV-barrier property, showing great potential to prevent ultraviolet induced lipid oxidation in food packaging [23,24].

However, the variable nature, chemical functionality and heterogeneity of technical lignin is an obstacle to further develop its material applications [25,26]. This is due to its source-dependent chemical structures [26,27] and the structural heterogeneity resulting from the fragmentation and condensation during the isolation process [28]. In order to solve this problem and improve lignin utilization in the materials field, several techniques have been developed to obtain homogenous lignin fractions. Historically, gel permeation chromatography [29], ultrafiltration [30,31] and selective precipitation [32,33] were applied to fractionate lignin into sub-fractions with improved homogeneity. More recently, renewed interest in fractionating lignin into homogeneous fractions using organic solvents has arisen [34,35]. These methods can be categorized into two groups: successive solvent extraction and incremental solvent extraction. As examples of the former group, Sun et al. fractionated soda-anthraquinone (AQ) lignin from the empty fruit bunches of oil palm by applying dichloromethane, n-propanol, and methanol-dichloromethane (7/3, *v*/*v*) in a successive manner, and found that the fraction with high molecular weight had high thermal stability [36]. A similar observation was also reported by another study on the fractionation of kraft-AQ lignin from Hardwood pellita with hexane, diethylether, methylene chloride, methanol, and dioxane [37]. Recently, a protocol using aqueous ethanol, acetone and propyleneglycol monomethyl ether was reported to be able to separate lignin into homogeneous fractions, even in the wet state [34]. For the latter group, incremental addition of a nonpolar solvent (hexanes) in a polar (acetone) solution was shown to produce narrow fractions from softwood wood kraft lignin [25].

Solvent extraction not only improves lignin’ homogeneity but also results in several lignin fractions with variable physicochemical properties [38], which were further found to influence their utilization in composite films. For instance, the antioxidant activity of the lignin fractions after successive solvent extractions has been demonstrated to improve the thermo-oxidative stability of prepared lignin-containing PU films [39]. When lignin fractions isolated from pine kraft lignin after solvent extraction were utilized to prepare starch-based composites, the fraction with low molecular weight was found to improve the mechanical properties of the films significantly, even though they were present in a small amount. This should originate from improved plasticity or adherence resulting from the interaction between the starch matrix and the low molecular weight fraction through hydrogen bonding, since the latter has more abundant carboxylic and phenolic hydroxyl polar groups [40,41].

Although some interesting results were obtained in previous studies, a better understanding of the impact of the source, molecular weight heterogeneity and chemical structure diversities of lignin on the performance of biocomposites is required. Therefore, the aim of present study is to understand the relationships between lignin structure (including molecular weight, number and types of functional groups) and performance in the 100% bio-based composite films (appearance, morphology, transparency, density, mechanical properties and thermal stability). In addition, based on the results obtained from this study, the specific lignin fractions are suggested to fulfil the required properties of the composite films, which is expected to facilitate lignin’s further development in the materials field.

## 2. Materials and Methods

### 2.1. Materials and Reagents

Softwood (Norwegian Spruce *Picea abies*) and Hardwood (*Eucalyptus Grandis*) lignin with moisture contents of 6.4% and 5.1% and ash contents of 0.6% and 1.2%, respectively, were obtained using the LignoBoost process [42]. The structural features of the lignin fractions were investigated and presented in our previous work [43]. Tunicate cellulose was purified from *Ciona intestinalis* using the prehydrolysis-kraft cooking-bleaching method [44]. The cellulose was subjected to enzymatic pretreatment followed by homogenization to prepare tunicate cellulose nanofiber (CNF). The obtained CNF was in the form of an aqueous suspension (0.5%, *w*/*w*), with a high crystallinity index (94%), a charge density of 43 μmol/g, a crystal size of 7.7 nm and an I_β_ ratio of 89.94% [45,46]. Commercial starch (water soluble, 80% amylopectin and 20% amylose, Sigma S-9765) with a molecular weight of 342.30 was purchased from Sigma-Aldrich, Stockholm, Sweden and used directly without treatment. All other non-specified reagents were of analytical grade, and they were purchased from VWR International AB, Stockholm, Sweden and used without additional treatment.

### 2.2. Successive Solvent Fractionation of Lignin

Successive solvent fractionation of lignin was conducted by modifying the method reported by Duval et al. [35]. The lignin was sequentially fractionated with ethyl acetate (EtOAc), ethanol (EtOH), methanol (MeOH) and acetone. In brief, 20 g of lignin samples (in dry weight) was dispersed in 200 mL of the specified solvent and stirred for 2 h. A Buchner filtration set with filter paper (Munktell, Sweden, Grade 3) was used to separate the soluble and insoluble fractions under vacuum. After filtration, another solvent was applied to re-disperse the insoluble fraction. This procedure was repeated to obtain various fractions. Finally, the soluble fractions were recovered using a rotary evaporator under reduced pressure, while the insoluble fractions were oven-dried at 40 °C under vacuum. See details in our previous study [43].

### 2.3. Preparation of Tunicate CNF-Starch-Lignin Films

Starch was dissolved in water (0.5%) under continuous stirring at 90 °C to obtain a clear solution, which was cooled to room temperature. Lignin was dissolved in acetone/water (4:1, *v*/*v*) with a concentration of 0.5%. 4 mL of the 0.5% tunicate CNF suspension prepared above, after blending with 4 mL starch solution and 2 mL lignin solution, was cast on Petri dishes to prepare films after drying at 50 °C overnight. The blank CNF-starch film was fabricated by mixing 4 mL CNF suspension and 4 mL starch solution, followed by the casting and drying conditions mentioned above. To facilitate discussion, the composite films were noted by their corresponding lignin source and the specific solvent used for fractionation, namely, SWEA, SWE, SWM, SWA and SWI for softwood lignin and HWEA, HWE, HWM, HWA and HWI for hardwood lignin, respectively. For example, SW and HW stand for softwood and hardwood, respectively, while EA, E, M, A and I indicate the ethyl acetate, ethanol, methanol, acetone and insoluble fraction, respectively.

### 2.4. Lignin Characterization

#### 2.4.1. Size-Exclusion Chromatography (SEC)

The molecular weight distributions of lignin were analyzed by size-exclusion chromatography (SEC) based on the methods reported by Guerra et al. [47]. The lignin fractions were acetobrominated before chromatographic analysis [48]. Briefly, a 5 mg lignin sample was firstly mixed with 900 μL glacial acetic acid and 100 μL of acetyl bromide. Then, the mixture was subjected to 2 h stirring. After acetobrominatation and the subsequent removal of excess glacial acetic acid and acetyl bromide, the acetobrominated lignin was dissolved in 1 mL tetrahydrofuran (THF, HPLC grade) and the resultant solution after filtering with a syringe filter (0.45 μm) was injected to SEC systems for molecular weight distribution analysis.

#### 2.4.2. ^31^P Nuclear Magnetic Resonance (^31^P NMR)

The functional groups of lignin were quantified by running ^31^P NMR [49]. A ~30 mg lignin fraction was dissolved in 100 µL dimethylformamide (DMF) and 100 µL of pyridine. The internal standard for quantification was 40 mg/mL of Endo-N-hydroxy-5-norbornene-2,3-dicarboximide (e-HNDI) (Sigma Aldrich) while the relaxation reagent was 5 mg/mL of chromium (III) acetylacetonate (Sigma Aldrich). Phosphorylation of lignin was achieved by adding 2-chloro-4,4,5,5-tetramethyl-1,3,2-dioxaphospholane, and then the lignin derivative was dissolved in CDCl_3_ before performing ^31^P NMR analysis.

#### 2.4.3. Pyrolysis-Gas Chromatography/Mass Spectrometry/Fire Ionization Detector (Py-GC/MS/FID)

A MicroDouble-shot Pyrolyser Py-2020iD (Frontier Lab, Fukushima, Japan) was connected with a Shimadzu GC/MS-QP 2010 apparatus (Kyoto, Japan) to conduct the Py-GC/MS/FID analysis. The separation was achieved by a capillary column RTX-1701 (Restec, Bellefonte, PA, USA) with dimensions of 60 m length, 0.25 mm width and 0.25 μm internal diameter. The pyrolysis was performed at 500 °C with a heating rate of 600 °C s^−1^. The other operation conditions and data analysis simply followed the ones reported previously [50].

### 2.5. Tunicate CNF-Starch-Lignin Films Characterization

#### 2.5.1. Density Measurement

A micrometer (NSK, Tokyo, Japan) was applied to determine the thickness of the films. The thicknesses at 20 points of each film specimen were measured and the mean value was used for density calculation. The density of the films was calculated based on weight, surface area and thickness and was reported as the mean value of at least three film specimens.

#### 2.5.2. Scanning Electron Microscope (SEM)

A Cressington 208HR high-resolution sputter coater (Watford, UK) was used to coat the composite films with gold. The thickness was controlled to be 3–5 nm. Then, a Field Emission SEM (Hitachi S-4800, Tokyo, Japan) was used to observe the morphological structures of the films.

#### 2.5.3. Transmittance Measurement

A Shimadzu UV-240 (Kyoto, Japan) was applied to determine the light transmission (T%) of the composite films. In order to compare different samples, the values were read at 650 nm. Film specimens were cut into a rectangular shape and then were loaded into the test cell. The transmittance values were used to evaluate the transparency of the films.

#### 2.5.4. Tensile Test

An Instron 4411 mechanical property tester (Instron Ltd., Norwood, MA, USA) was applied to analyze the mechanical performance of the films. The measurement was conducted with a 20 mm starting grip gap and a 3 mm/min grip moving rate. The test was performed on at least 3 specimens for each film and the mean values were reported.

#### 2.5.5. Thermal Gravimetric Analysis (TGA)

A Metler Toledo Star TOA/SDTA 851*e* device (Columbus, OH, USA) was applied to conduct thermal analysis. In this study, both nitrogen and oxygen atmospheres were tested under a gas flow rate of 50 mL min^−1^. The analysis was performed between 20 and 800 °C with a heating rate of 10 °C min^−1^. The maximum decomposition temperatures were determined by the first derivative TGA curve (DTG).

## 3. Results

### 3.1. Lignin Diversities after Successive Solvent Extraction

When successive solvent fractionation with ethyl acetate, ethanol, methanol and acetone was applied to both softwood and hardwood lignin, five fractions with different yields (Table 1) were recovered from each lignin. For softwood lignin, the most abundant fraction was recovered by ethanol with a yield of 32.5%. This was followed by ethyl acetate (24.5%), acetone (14.7%) and methanol (8.2%). Finally, the insoluble fraction weighed 20%. Similarly, the hardwood lignin extraction resulted in fractions with higher yields from ethyl acetate (35.2%) and ethanol (32.8%). However, the insoluble fraction had a lower yield of 10.5%, compared to 20% for hardwood lignin.

The SEC curves of the lignin fractions are shown in Figure S1 and the corresponding molecular weight distribution data is summarized in Table 1. The initial softwood lignin had a *M*_n_ of 1580 and a *M*_w_ of 5440, higher than the corresponding values of 910 and 1740 for the initial hardwood lignin. However, softwood lignin showed a much broader molecular weight distribution than hardwood lignin, as indicated by its higher polydispersity index (PDI = 3.5). As seen in Table 1, the weight average (*M*_w_) and number average (*M*_n_) molecular weights of each fraction after successive solvent extraction increased along the fractionation procedure, due to the increased dissolving capacity, regardless of lignin source. EtOAc-soluble fractions from both softwood and hardwood lignin had relatively low M_n_ and *M*_w_, implying that they were mainly composed of oligomeric lignin derivatives [51]. The insoluble fractions showed generally higher molecular weight than that of the initial lignin, particularly for softwood lignin. This is likely due to the presence of noticeable quantities of carbohydrates that can be covalently bonded to lignin forming lignin–carbohydrates complexes [52]. All solvent soluble fractions had lower PDI than the initial lignin, suggesting the improved homogeneity. This agrees well with previous conclusions that successive solvent extractions could produce more homogenous lignin [37]. In general, the softwood lignin fractions had higher molecular weights than the corresponding fractions for hardwood lignin when the same solvent was applied. In addition, the fractions with higher molecular weight had a higher PDI, whereas those with lower molecular weight had a lower PDI, which agrees well with other studies [16,25]. Compared to the low PDI of 1–2 for the soluble fractions, the insoluble fractions had much higher PDIs, with 6.2 and 4.82 for softwood and hardwood lignin, respectively. This indicated that the insoluble fractions had higher structural heterogeneity than the soluble fractions.

**Figure 1:**
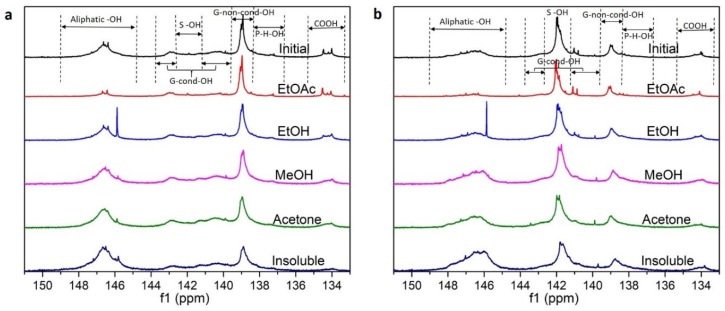
Signal assignment of quantitative ^31^P NMR spectra for softwood (**a**) and hardwood (**b**) lignin fractions.

^31^P NMR technique is used to analyze the contents of the functional groups in the lignin fractions. The obtained NMR spectra and the signal assignment are shown in Figure 1. Based on ^31^P NMR results, the contents of aliphatic –OH, –COOH and phenolic –OH groups of lignin fractions are calculated and summarized in Table 1. In general, with successive solvent extraction, the aliphatic –OH group content increased, while –COOH and phenolic –OH groups contents decreased regardless of the lignin source. In general, the lower molecular weight fractions always have less aliphatic –OH groups and more phenolic –OH groups. This might be due to the kraft process in which the native lignin was degraded into kraft lignin with relatively small molecular weight by forming new phenolic –OH groups and cleaving aliphatic –OH groups [51].

**Figure 2:**
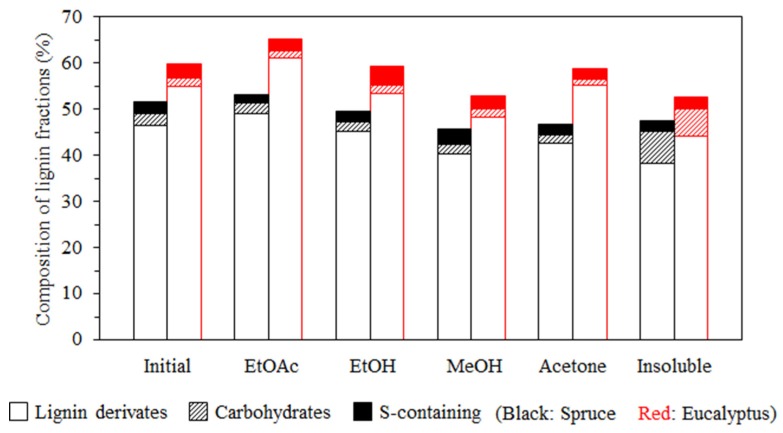
Composition of spruce and eucalyptus lignin fractions in terms of lignin derivates, carbohydrates and S-containing compounds, as determined by Py-GC/MS/FID.

As an important analytical technique to investigate the complex structures of insoluble polymers, Py-GC/MS/FID method is also applicable to lignin analysis [53,54,55,56]. The composition of lignin fractions analyzed by Py-GC/MS/FID is presented in Table S1 and simply summarized in Figure 2 in terms of the relative contents of lignin derivatives, carbohydrate derivatives and sulfur-containing (S-containing) compounds. As seen in Figure 2, lignin is the main component of all fractions, while the content of the carbohydrates varied between 2% and 7%, and the highest amounts are present in insoluble fractions for both softwood and hardwood lignin. This is most likely due to the presence of lignin–carbohydrate complexes in these fractions [31]. The relative content of sulfur-containing compounds varied insignificantly between the two types of lignin and between different fractions. The ethanol and methanol fractions appeared to be enriched with sulfur-containing compounds in the hardwood and softwood samples, respectively.

To determine the structural diversities in the lignin fractions, the products of lignin after pyrolysis were categorized based on their side-chain and the ortho-substitution in aromatic ring (Table 2). The detailed calculation methods for contents of the specific structural features, including methoxylated lignin units, double bonds in the Cα positions and units with short side chains ((ArC_1_ + ArC_2_)/ArC_3_), can be found in our previous work [43]. As shown in Table 2, the hardwood lignin fractions had a higher content of the syringyl (S) and quiacyl (G) units. The EtOAc fraction for hardwood lignin contained a higher number of methoxy groups, while other structures had a similar lower content. In softwood lignin, no clear correlation was observed. EtOAc fractions for both types of lignin had significantly different values for each type of phenol, especially in hardwood lignin. It had a lower amount of saturated side chain-containing phenols, a lower content of α-double bond-containing phenols, and the highest content of oxygen present in a side chain (such as vanillin and coniferyl alcohol, etc.), with the highest ratio of phenols with a shortened side chain.

### 3.2. Impact of Lignin on Film Appearance and Morphology

The present study is focused on investigating the impacts of distinct fractions recovered from kraft lignin from different sources (both softwood and hardwood) by sequential solvent extraction on the performance of the corresponding 100% bio-based tunicate cellulose nanofibers (CNF)-starch-lignin composite films. In fact, green composite films prepared from cellulose, starch and lignin were reported by Wu et al. [57], who used the ionic liquid (IL), 1-allyl-3-methylimidazolium chloride (AmimCl), to completely dissolve these three components. However, during dissolution, the cellulose lost its inherent crystallinity, and the reinforcement effect was no longer present. In addition, the ionic liquid is costly and impractical for large scale applications. To overcome this drawback, we developed an aqueous system to mix cellulose, starch and lignin. Moreover, tunicate CNF with high crystallinity, large crystal size, high molecular weight, large aspect ratio and excellent thermal stability [44,45,46], rather than woody cellulose as a reinforcement, was introduced to improve the performance of the composite films.

After successive solvent extraction was applied to lignin from different sources (both softwood and hardwood), eight soluble fractions and two insoluble fractions were successfully isolated. All fractions showed great diversities in terms of molecular weight distribution and chemical structures, which is expected to lead to different impacts when they are utilized to fabricate composite materials. To investigate this, we chose tunicate CNF-starch composite film as the blank, and then films containing identical amount of different lignin samples were prepared and characterized. As shown in Figure S8, the introduction of lignin to the CNF-starch composite films is verified by the presence of the peak 1515 cm^−1^ for all the lignin-containing composite films, which generates from the asymmetric aryl ring stretching in lignin [58]. The interrelation between lignin properties and film performance was further studied.

**Figure 3:**
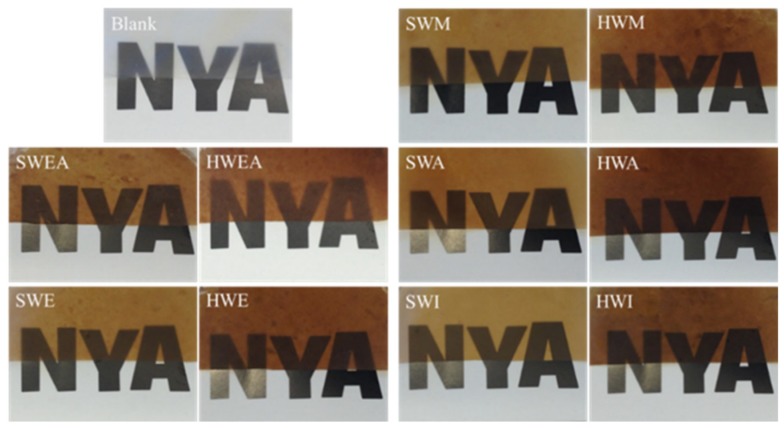
Digital photos of CNF-starch film and composite films containing different lignin fractions of spruce and eucalyptus lignin.

**Figure 4:**
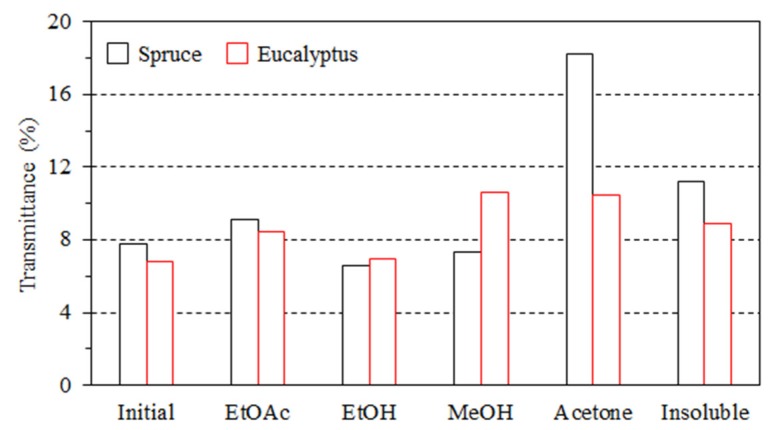
Transmittance of composite films containing different fractions of spruce and eucalyptus lignin.

**Figure 5:**
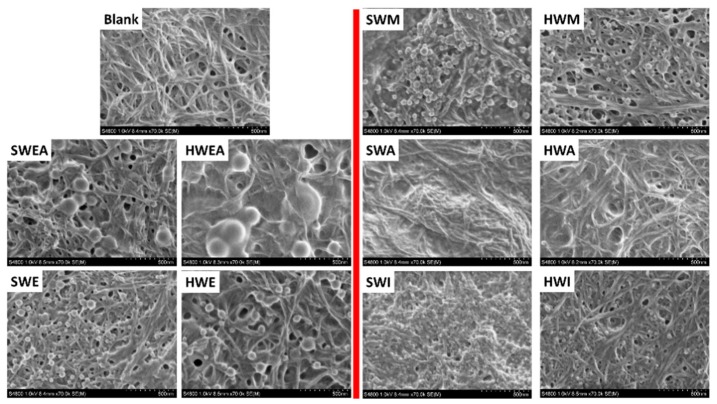
SEM images of CNF-starch film and composite films containing different lignin fractions.

As seen in Figure 3 and Figure 4, the blank CNF-starch film was colorless having a high transmittance of 46% and a thickness of 11.8 µm (Table S2) when lignin was absent. SEM observations showed that the fibrillary net-like structure was formed from self-assembly of CNF, although 50% starch was added (Figure 5). However, pore structures were observed because the fibrillary nature of CNF was still obvious. We speculate that CNF and starch formed a core–shell structure, namely, that the CNF fibril is the core covered by a starch shell, as previously reported by Prakobna et al. [59]. In fact, this CNF-starch film is different from the normally reported cellulose-starch composites, in which the starch is a major matrix, while small amounts of cellulose fibres were introduced as reinforcement [60].

When lignin was introduced, the prepared composite films became thicker (thickness of 12.8–16.3 µm, Table S2) and yellowish irrespective of the lignin source and fraction. This is due to the inherent brownish colour of the lignin. Correspondingly, the transmittances of the composite films were quite low, ranging from 6% to 18%. However, composite films with hardwood lignin showed a deeper color than those with softwood lignin, which might be partially due to the poorer distribution of hardwood lignin in the composite films than softwood lignin, as indicated by some visual aggregates (Figure 3). For all composite films except SWA and HWA, lignin aggregated to form particles during film drying. The formation of lignin particles within a few micrometers in starch-based films is regarded as a result of poor compatibility between lignin and starch [40,41]. Additionally, the lignin particle formation was also related to the solubility of lignin and evaporation process during film preparation. Generally, all lignin fractions could be dissolved in acetone/water (4:1, *v*/*v*). However, when lignin solution was mixed with starch and cellulose in water, the acetone/water ratio decreased, and lignin was no longer soluble, resulting in aggregation. In addition, during evaporation, the acetone was easier to remove than water, and the poor solubility of lignin in water also contributed to the lignin particle formation. Irrespective of the source, lignin fractionated with EtOAc formed the biggest particles, with 100–200 nm diameter, followed by EtOH and MeOH-extracted lignin (particles with 50–100 nm in diameter) and then the insoluble fractions (mainly <100 nm). Interestingly, those extracted with acetone showed no visual particle formation. This finding agreed well with the finding mentioned above that SWA and HWA had highest transmittances of 18.22% and 10.47% for softwood and hardwood fractionated lignin, respectively (Figure 4). This might originate from their best solubility in acetone compared to other fractions. Moreover, the size of the formed lignin particles in the composite films has been found to affect their contact angles. As shown in Table S3, SWA and HWA without lignin aggregation had the highest contact angles of 71.9° and 68.9° for softwood and hardwood fractionated lignin, respectively. In contrary, SWEA and HWEA with the biggest lignin particles showed the lowest contact angles, 45.8° and 50.5°, respectively.

### 3.3. Impact of Lignin on Film Mechanical Properties

**Figure 6:**
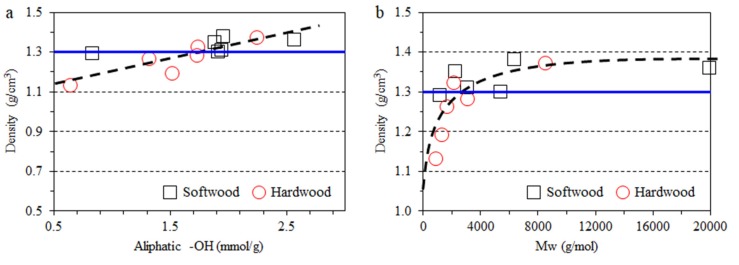
Relationship of film density to aliphatic –OH group content (**a**) and *M*_w_ (**b**) of lignin (Blue line: Blank film).

**Figure 7:**
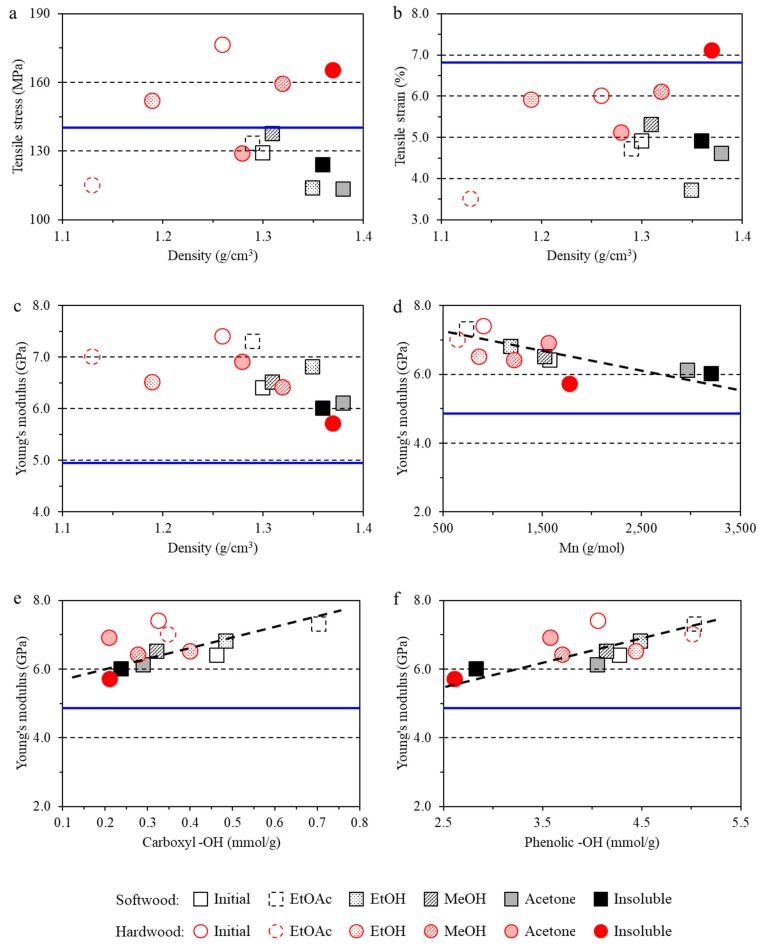
Relationship of mechanical properties of composite films to film density (**a**–**c**) and *M*_n_ (**d**), carboxyl –OH group content (**e**) and phenolic –OH group content (**f**) of lignin. (Blue line: Blank film).

Higher aliphatic –OH group content of lignin is correlated with denser composite film (Figure 6a). This may originate from the newly formed hydrogen bonds between the aliphatic –OH of lignin and other –OH groups from both cellulose and starch. Moreover, film density is related to lignin *M*_w_ (Figure 6b). The composite film became denser when the lignin *M*_w_ increased up to ~5000. However, the film’s density was stable when *M*_w_ was above 5000. No clear correlation between the density and mechanical performance of films was observed (Figure 7a–c). However, the lignin source was found to be critical to the mechanical performance of the films. In general, compared to the CNF-starch film, the hardwood lignin addition resulted in higher tensile stress, while softwood lignin lowered the tensile stress of the composite films (Figure 7a). This difference might be related to the uniformity of the lignin dispersity in the composite films. As observed in Figure 3, some aggregates could even be observed by the naked eye in the composite films containing hardwood lignin, which may explain the lower tensile stress. In addition, the introduction of lignin was found to induce lower tensile strain and higher Young’s modulus irrespective to lignin species or fractions (Figure 7b,c). This finding agrees well with the previous conclusion that lignin is inherently a rigid polymer [41].

Young’s modulus of the films was related to the molecular weight (*M*_n_) of the lignin (Figure 7d). Higher molecular weight was correlated with lower Young’s modulus. The small molecular weight lignin could act as a compatibilizing agent between starch and CNF in the composite films, thus improving the integrity of the films and improving mechanical properties. In contrast, the poor miscibility of large molecular weight lignin with the matrix in the composite films resulted in lower mechanical strength [41]. Our results agree well with these observations. The chemical structure of lignin, especially the functional groups, was also important to the mechanical properties of the films (Figure 7e,f). More carboxyl –OH and phenolic –OH groups existing in the lignin structure could enhance hydrogen bond formation between lignin and the other two components containing abundant –OH groups, thus reinforcing the network structure as indicated by increased Young’s modulus.

### 3.4. Impact of Lignin on Film Thermal Stability

TGA was performed in both nitrogen and oxygen atmospheres to assess the thermal and thermo-oxidative stabilities of the films with lignin addition, respectively, and to evaluate the relation of lignin addition to the thermal properties of these composite films.

All TGA curves of composite films in nitrogen atmosphere were presented in Figures S2 and S3. The starting decomposition temperature (*T*_5%_) corresponding to a 5% weight loss and the maximum decomposition temperature (*T*_max_) were chosen to evaluate the films’ thermal stability. As shown in Table 3, the blank CNF-starch film showed a *T*_5%_ of 283.3 °C, a *T*_max_ of 355 °C and a residual weight (char) of 10.3% at 800 °C. Lignin addition has been found to influence the thermal stability of the composite films negatively, as indicated by lower *T*_5%_ (265–278 °C), though the softwood lignin was more thermally stable than hardwood lignin. The addition of both softwood and hardwood lignin did not significantly affect the *T*_max_ of the composite films (354–360 °C). However, the char contents for all composite films (12–19%) were higher than for the blank CNF-starch film (10.3%). The high char formation was due to the lignin which was abundant in aromatic rings with various branches, and these complex and highly condensed structures could result in a high amount of unvolatized residue at 800 °C [27]. With respect to source, the addition of softwood lignin seemed to produce a higher content of char than hardwood lignin. In general, char formation was found to increase as molecular weight increases. The composite films containing lignin with smallest molecular weights, namely, EtOAc-soluble fractions for both softwood and hardwood lignin, showed the lowest char content, while the addition of the highest molecular weight lignin (insoluble fractions) resulted in highest char content.

**Figure 8:**
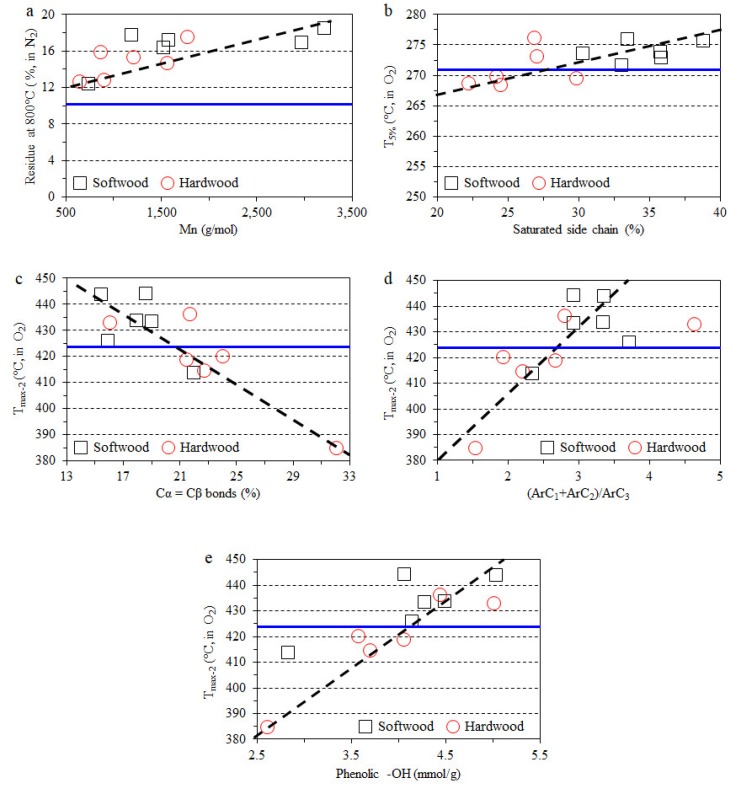
Correlation between lignin properties and composite films. (**a**) correlation between *M*_n_ of lignin and residue at 800 °C of films; (**b**) correlation between saturated side chain of lignin and *T*_5%_; (**c**–**e**) correlations between Cα = Cβ bond content, shortened side chain ratio and phenolic –OH group content of lignin and *T*_max2_ of films. (Blue line: Blank film).

The thermo-oxidative stability of lignin-containing composite films was further measured in an oxygen atmosphere [61]. All TGA curves in oxygen are presented in Figures S4 and S5. The comparison between DTG curves of identical composite film (Figures S6 and S7) in both nitrogen and oxygen atmosphere exhibited significant differences, namely, only one peak for the maximum degradation temperature (*T*_max_) for the former and two peaks of *T*_max_ (*T*_max1_ and *T*_max2_) for the latter. This phenomenon suggests a difference between the mechanism of thermal degradation in oxygen and in nitrogen. As shown in Table 3, the blank CNF-starch film showed a *T*_5%_ of 270.8 °C, a *T*_max1_ of 325 °C and a *T*_max2_ of 424 °C, in the oxygen atmosphere. The addition of softwood lignin resulted in higher *T*_5%_ (271–276 °C) for the composite films. However, the introduction of some hardwood lignin fractions, such as EtOAc-soluble and insoluble lignin, even lowered the *T*_5%_. The residue at 800 °C, namely, ashes, weighed 0.8% for blank film. The composite films showed quite similar ash contents at 800 °C to the blank film. In general, the composite films containing softwood lignin showed higher *T*_max2_ than those with hardwood lignin. Furthermore, the chemical structures of lignin exhibited significant influences on the thermo-oxidative stabilities of the composite films in oxygen. As seen in Figure 8, the lignin with a higher amount of phenols containing saturated side chains led to a higher *T*_5%_ of the corresponding composite film in oxygen. This finding indicated that more saturated side chains could provide benefit to the thermos-oxidative stability of the lignin-containing films. In addition, as the content of phenols having α-double bonds increased, *T*_max2_ decreased. However, the *T*_max2_ increased when increasing (ArC_1_ + ArC_2_) per ArC_3_, until the latter reached approximately 3. *T*_max2_ was also positively related to the phenolic –OH group content of the lignin, which was also observed in polypropylene-lignin composite films [62].

## 4. Conclusions

- In this study, the lignin fractions with great diversities have been found to affect the properties of the tunicate CNF-starch composite films significantly:
  - The lignin aggregated to form particles in the composites, thus affecting the color and transmittance value.
  - The lignin imparted to the films a lower tensile strain and higher Young’s modulus due to the inherent rigidity of lignin. However, hardwood lignin with lower molecular weight and polydispersity reinforced the film structure, while softwood lignin with a higher molecular weight and lower G+S unit content negatively affected the tensile stress of the films.
- The thermal stability of composite films in a nitrogen atmosphere have a clear positive correlation with the molecular weight of lignin, and the char formation was improved with the addition of the lignin fraction.
- The direct relationship between the certain lignin structural features and thermo-oxidative stability of lignin-containing composite films was also observed. The same structural features positively influence the antioxidant properties of lignin.
- The structure–performance relationships found here will help to tune the properties of lignin to fit in specific application in the materials field. For example, as found in this study, the acetone lignin fraction is the best candidate to prepare uniform and highly transparent composite films; insoluble lignin fraction with high molecular weight could render the composite film dense structure; the ethyl acetate lignin fraction with low molecular weight, low PDI, and high content of phenolic –OH group are the best for composite films to have high Young’s modulus and good thermal stability.

## Figures and Tables

**Table 1:** Yield, molecular weight properties and functional group content of lignin *.

Type	Fraction	Yield (%)	Mn (g/mol)	Mw (g/mol)	PDI	Aliph –OH (mmol/g)	–COOH (mmol/g)	Ph –OH (mmol/g)
Spruce	Initial	-	1490	6650	4.48	1.93	0.46	4.27
	EtOAc	24.5	740	1200	1.63	0.85	0.67	5.10
	EtOH	32.5	1190	2280	1.93	1.91	0.48	4.60
	MeOH	8.2	1530	3080	2.01	1.96	0.31	4.15
	Acetone	14.7	2970	6360	2.14	1.95	0.27	4.02
	Insoluble	20.0	3210	19,930	6.20	2.76	0.21	3.04
Eucalyptus	Initial	-	900	1980	2.20	1.33	0.32	4.23
	EtOAc	35.2	650	950	1.47	0.65	0.35	5.02
	EtOH	32.8	870	1390	1.60	1.52	0.40	4.45
	MeOH	15.4	1220	2170	1.78	1.74	0.28	3.71
	Acetone	6.1	1570	3150	2.00	1.74	0.21	3.58
	Insoluble	10.5	1780	8570	4.82	2.25	0.21	2.62

* including data cited from previous study [43].

**Table 2:** Phenol distribution of lignin as analyzed by Py-GC/MS/FID.

Type	Fraction	G + S Units %	Saturated Side Chain %	Cα = Cβ Bonds %	O-Atoms in Side Chain %	(ArC_1_ + ArC_2_)/ArC_3_ *
Spruce	Initial	83.8	33.5	19.0	10.9	2.9
	EtOAc	84.6	30.3	15.4	12.5	3.4
	EtOH	84.6	35.8	18.0	9.6	3.4
	MeOH	82.2	38.8	15.9	9.8	3.7
	Acetone	82.7	35.8	18.6	11.7	2.9
	Insoluble	85.3	33.1	22.0	13.3	2.3
Eucalyptus	Initial	93.6	24.3	21.5	15.7	2.7
	EtOAc	94.0	24.5	16.1	20.5	4.6
	EtOH	92.6	29.9	21.7	10.0	2.8
	MeOH	92.7	27.1	22.8	9.2	2.2
	Acetone	92.3	26.9	24.1	11.5	1.9
	Insoluble	92.9	22.3	32.1	12.0	1.5

* ArC_1_, ArC_2_, and ArC_3_ are Py-products with 1, 2, and 3 carbons in their side chains, respectively (%).

**Table 3:** Decomposition temperatures and residues at 800 °C of composite films analyzed by TGA in both nitrogen and air atmospheres.

Type	Fraction	TGA (N_2_)			TGA (O_2_)			
		*T*_5%_	*T*_max_	Residue at 800 °C (char)	*T*_5%_	*T*_max-1_	*T*_max-2_	Residue at 800 °C (ash)
		°C	°C	%	°C	°C	°C	%
Blank		283.3	354.7	10.3	270.8	325.2	423.6	0.8
Spruce	Initial	270.8	354.8	17.2	275.8	319.0	433.2	0.5
	EtOAc	266.7	359.9	12.4	273.5	320.1	443.8	0.6
	EtOH	274.2	355.2	17.7	272.8	321.3	433.5	0.5
	MeOH	272.8	354.2	16.4	275.7	321.5	425.9	0.9
	Acetone	277.5	353.7	16.9	273.8	321.2	444.1	0.6
	Insoluble	271.2	355.3	18.5	271.7	326.2	413.5	0.8
Eucalyptus	Initial	268.7	358.7	12.7	269.7	319.2	418.6	0.7
	EtOAc	265.2	359.7	12.6	268.3	318.9	432.8	0.5
	EtOH	265.7	355.9	15.8	270.5	311.4	439.4	0.4
	MeOH	270.7	354.8	15.2	273.0	326.7	414.4	0.8
	Acetone	275.7	356.2	14.6	276.0	326.1	419.9	0.8
	Insoluble	268.8	357.6	17.4	268.5	331.3	384.4	1.3

## Supplementary Materials

The following are available online at https://www.mdpi.com/2073-4360/11/3/538/s1, Table S1: Composition and specific structural features of lignin fractions (by Py-GC/MS-FID), Table S2: Film thickness (µm) of all lignin-CNF-starch composite films and blank films, Table S3: Contact angle (°) of all lignin-CNF-starch composite films and blank films, Figure S1: Molecular weight distribution of lignin fractions from softwood (a) and hardwood (b), Figure S2: The TGA curves of composite films containing softwood lignin fractions (N_2_ atmosphere), Figure S3: The TGA curves of composite films containing hardwood lignin fractions (N_2_ atmosphere), Figure S4: The TGA curves of composite films containing softwood lignin fractions (O_2_ atmosphere), Figure S5: The TGA curves of composite films containing hardwood lignin fractions (O_2_ atmosphere), Figure S6: The DTG curves of composite films containing spruce lignin fractions (both N_2_ and O_2_ atmosphere), Figure S7: The DTG curves of composite films containing eucalyptus lignin fractions (both N_2_ and O_2_ atmosphere), Figure S8: FTIR spectra of all lignin-CNF-starch composite films and blank films.
