# Supplementary material for: The Impact of Lignin Structural Diversity on Performance of Cellulose Nanofiber (CNF)-Starch Composite Films
Source: Polymers (Basel). 2019 Mar 21;11(3):538. doi: 10.3390/polym11030538 (PMC6473382; doi:10.3390/polym11030538)
Supplement: Supplementary file 1 [file polymers-11-00538-s001.pdf]

Supporting Information for publication

Lignin-modified tunicate cellulose nanofiber (CNF)-starch composites: impact of lignin diversity on film performance

Ayumu Tagam [a,b], Yadong Zhao[a], Galina Dobele[c], Mikael E. Lindström[a] and Olena Sevastyanova[a,d*]*

[a] Department of Fibre and Polymer Technology, KTH, Royal Institute of Technology, Teknikringen 56-58, 100 44 Stockholm, Sweden.

[b] Research Laboratory, Nippon Paper Industries Co., Ltd., 5-21-1 Oji, Kita-ku, Tokyo 114-0002, Japan

[c] Latvian State Institute of Wood Chemistry, 27 Dzerbenes Str. LV 1006 Riga, Latvia

[d] WWSC, KTH Royal Institute of Technology, Teknikringen 56-58, Stockholm, 100 44, Sweden

Number of Pages: 8

Number of Tables: 3

Number of Figures: 8

Table S1. Composition and specific structural features of lignin fractions (by Py-GC/MS-FID)

			Initial	EtOAc	EtOH	MeOH	Acetone	Insoluble
Spruce	Carbohydrates (C)	%, from chromatogram	2.5	2.5	2.0	2.1	1.7	7.0
	Lignin derivates (L)		46.4	48.9	45.1	40.2	42.6	38.3
	Summary: C+L = 100%		48.9	51.4	47.1	42.3	44.3	45.2
	Summary: Carbon dioxide, Methanal, Water, Methanol		46.7	43.9	47.4	52.4	51.9	51.2
	S-containing		2.7	1.8	2.3	3.3	2.3	2.3
	Carbohydrates (C)	%, from C+L	5.1	4.8	4.3	4.9	3.9	15.4
	Acid, Ester	%, from C	26.2	23.5	31.9	31.6	39.7	20.5
	Aldehyde, Ketone		21.0	13.0	16.7	10.1	21.3	39.2
	Cyclopentane derivates		28.6	45.8	27.9	21.5	20.1	9.6
	Furan		21.8	17.8	22.6	34.0	18.4	21.4
	Pyran		2.4	0	1.0	2.9	0.6	4.2
	Sugars		0	0	0	0	0	5.2
	Lignin derivates (L)	%, from C+L	94.9	95.2	95.7	95.1	96.1	84.6
	P and B derivates	%, from L	16.2	15.4	15.4	17.8	17.3	14.7
	G derivates		83.8	84.6	84.6	82.2	82.7	85.3
Eucalyptus	Carbohydrates (C)	%, from chromatogram	2.0	1.5	1.8	1.7	1.3	5.9
	Lignin derivates (L)		54.8	61.2	53.4	48.2	55.1	44.1
	Summary: C+L = 100%		56.8	62.6	55.1	50.0	56.4	50.0
	Summary: Carbon dioxide, Methanal, Water, Methanol		39.3	32.4	39.6	46.2	40.5	46.2
	S-containing		3.0	2.5	4.2	2.8	2.3	2.7
	Carbohydrates (C)	%, from C+L	3.4	2.4	3.2	3.5	2.3	11.7
	Acid, Ester	%, from C	35.9	57.1	47.7	44.5	54.3	22.0
	Aldehyde, Ketone		14.9	12.1	16.5	16.8	18.6	31.7
	Cyclopentane derivates		14.9	17.5	15.9	15.0	14.7	7.4
	Furan		16.4	9.4	11.9	21.4	12.4	22.4
	Pyran		2.6	0	0.6	2.3	0	10.8
	Sugars		15.4	4.0	7.4	0	0	5.8
	Lignin derivates (L)	%, from C+L	96.6	97.6	96.8	96.5	97.7	88.3
	P and B derivates	%, from L	3.5	3.6	3.9	3.5	4.3	3.1
	G derivates		27.3	24.9	28.7	28.7	31.7	35.7
	S derivates		66.3	69.1	63.9	64.1	60.6	57.1
	G+S derivates		93.6	94.0	92.6	92.7	92.3	92.9

Table S2. Film thickness (μm) of all lignin-CNF-starch composite films and blank films

	Initial	EtOAc	EtOH	MeOH	Acetone	Insoluble
Softwood	13.5 ± 0.7	14.4 ± 0.7	12.8 ± 0.8	14.1 ± 0.8	16.3 ± 0.8	13.6 ± 0.5
Hardwood	13.6 ± 1.1	14.4 ± 0.7	13.8 ± 0.4	13.5 ± 0.9	14.3 ± 0.8	13.9 ± 0.8
Blank			11.8 ± 1.4			

Table S3. Contact angle (°) of all lignin-CNF-starch composite films and blank films

	Initial	EtOAc	EtOH	MeOH	Acetone	Insoluble
Softwood	66.2	45.8	56.7	66.1	71.9	60.1
Hardwood	60.0	50.5	59.4	61.9	68.9	53.1
Blank			57.0			

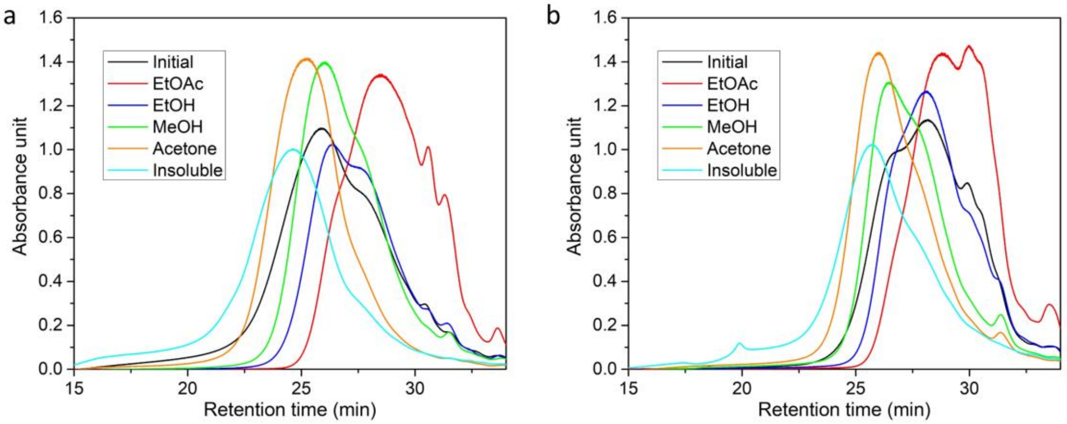

Figure S1. Molecular weight distribution of lignin fractions from softwood (a) and hardwood (b)

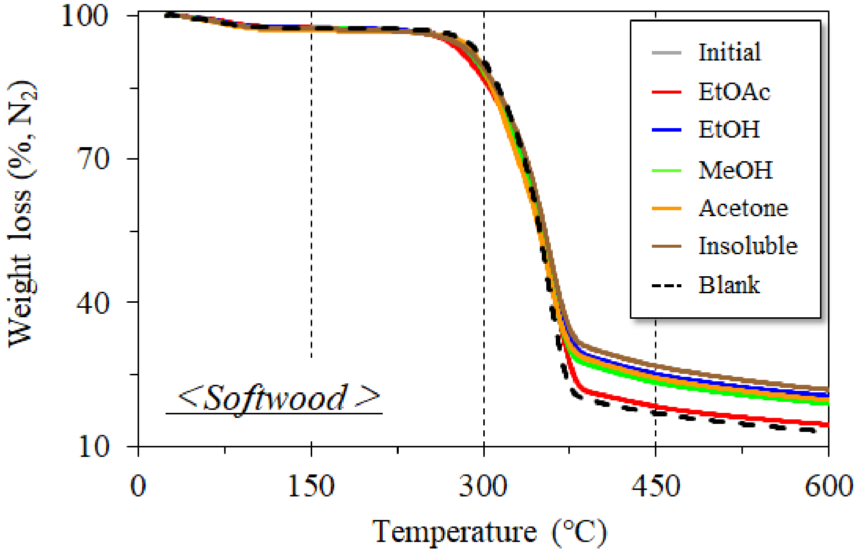

Figure S2. The TGA curves of composite films containing softwood lignin fractions (N_2 atmosphere)

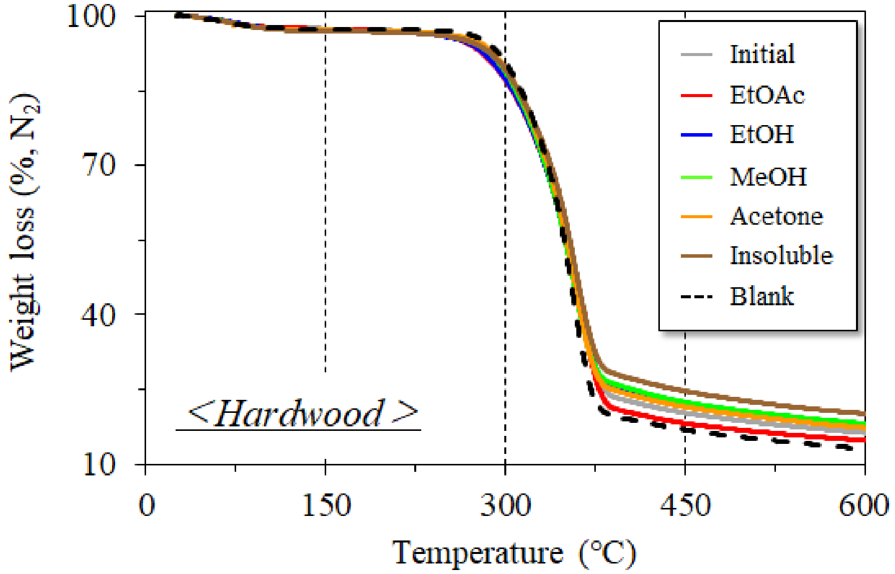

Figure S3. The TGA curves of composite films containing hardwood lignin fractions (N_2 atmosphere)

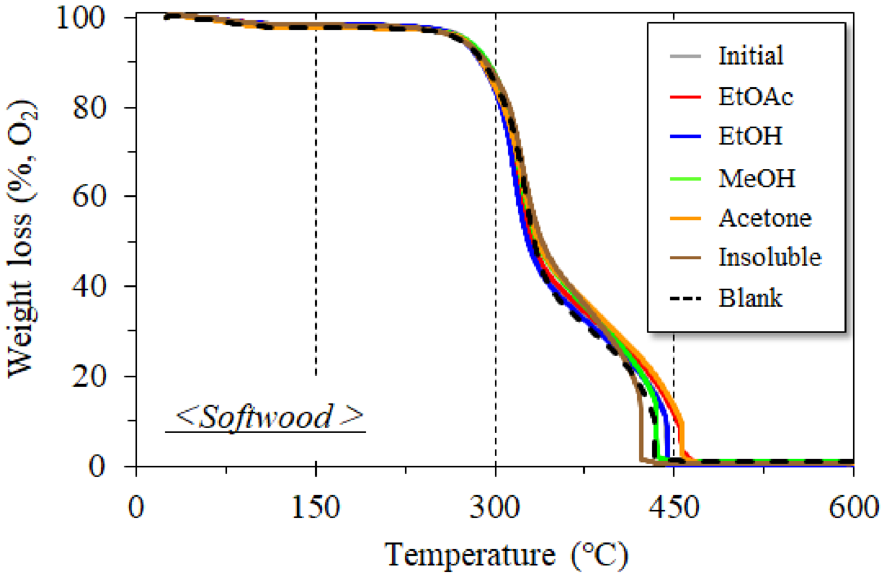

Figure S4. The TGA curves of composite films containing softwood lignin fractions (O_2 atmosphere)

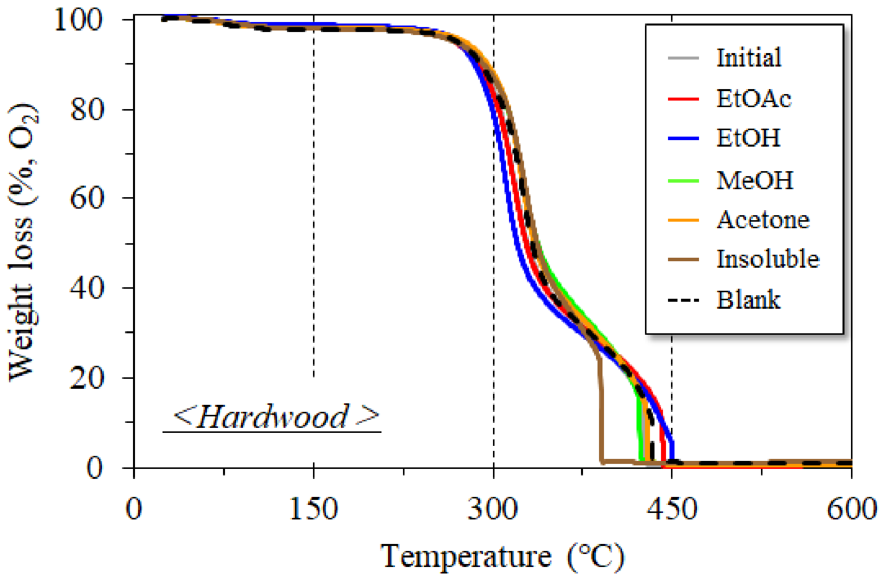

Figure S5. The TGA curves of composite films containing hardwood lignin fractions (O_2 atmosphere)

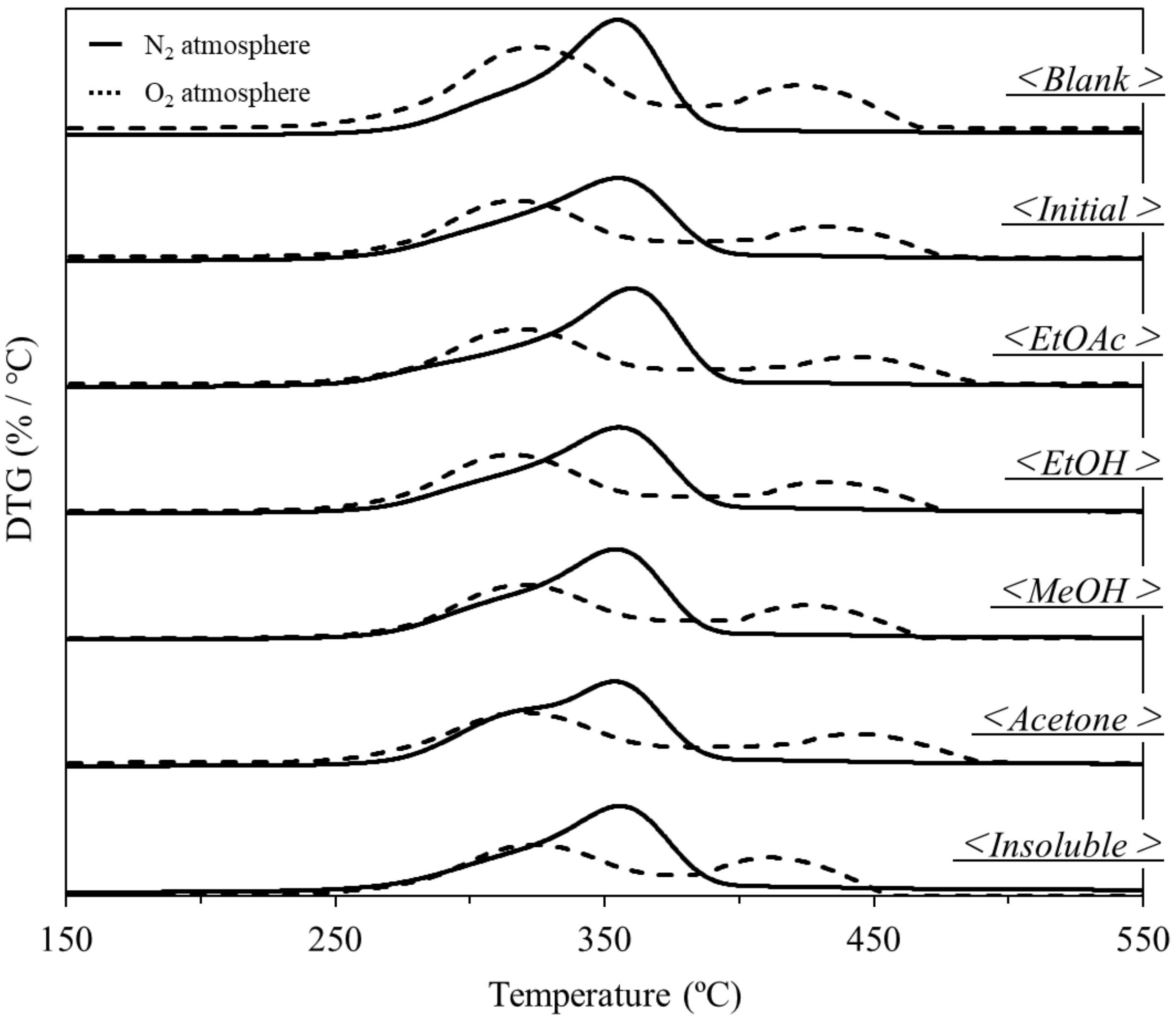

Figure S6. The DTG curves of composite films containing spruce lignin fractions (both N_2 and O_2 atmosphere)

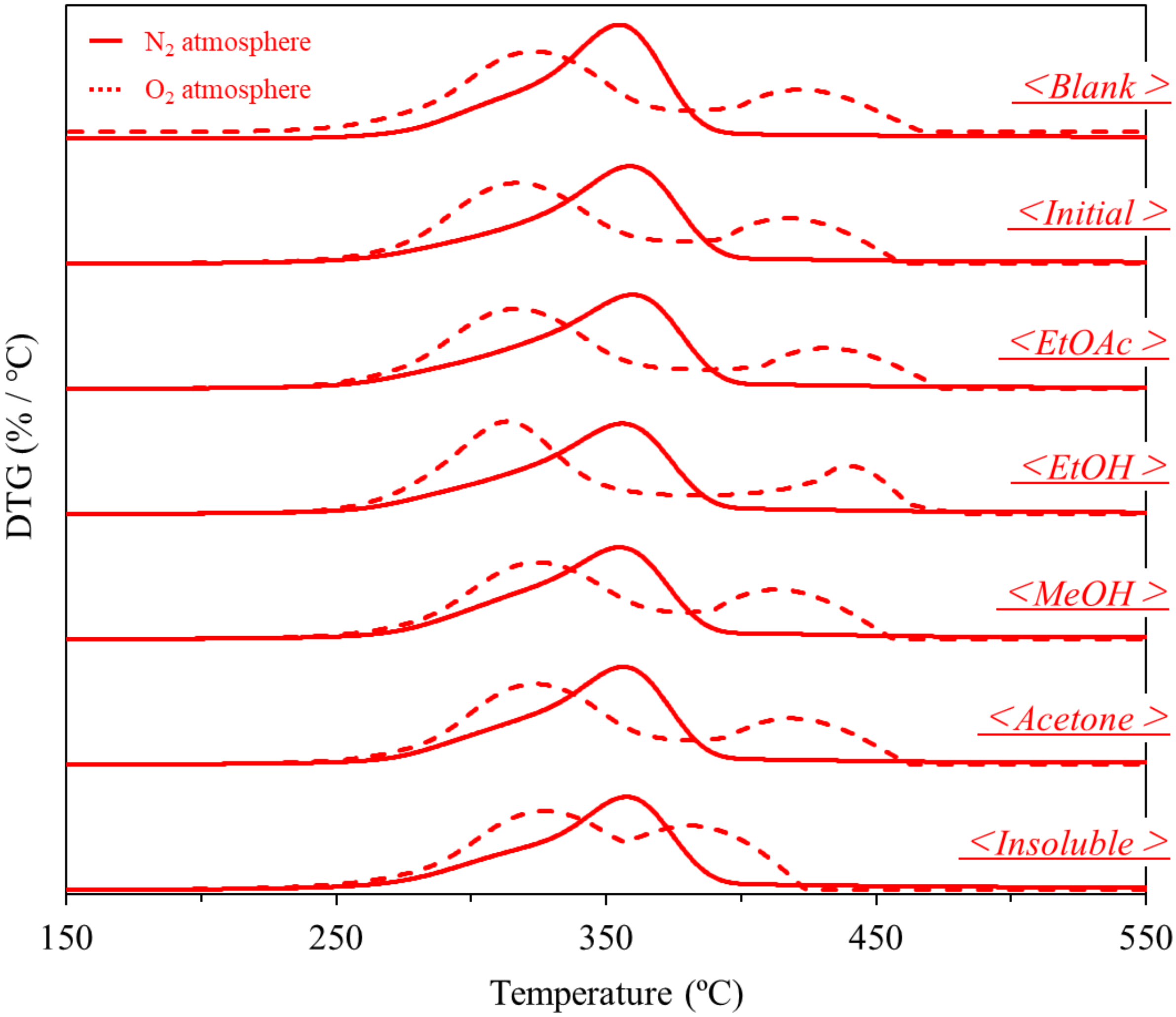

Figure S7. The DTG curves of composite films containing eucalyptus lignin fractions (both N_2 and O_2 atmosphere)

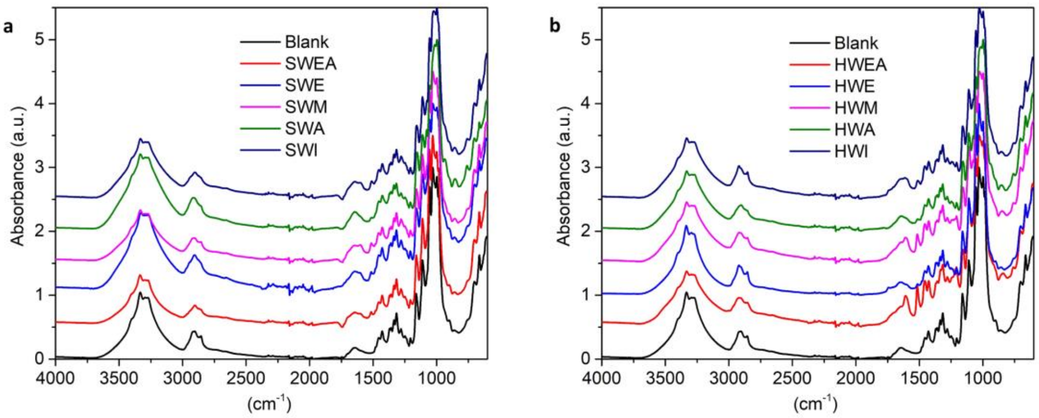

Figure S8. The FTIR spectra of all lignin-CNF-starch composite films and blank films
